# A case report of acute dermatitis that developed during an experiment examining the bromination of 3-hexylthiophene

**DOI:** 10.1186/1745-6673-5-3

**Published:** 2010-02-27

**Authors:** Mikiya Sato, Hajime Yoshiki, Masaki Horie, Eiji Yano

**Affiliations:** 1 Teikyo University School of Medicine, Department of Hygiene and Public Health, Japan; 2 Kawakita General Hospital, Centre for Family Practice, Tokyo, Japan; 3 Riken, Safety Division, Japan; 4 The University of Manchester, UK; 5 Riken, Health Center, Japan

## Abstract

Occupational cases with allergic reaction to fragrance substances, which refer to various chemicals providing aroma characteristics, are arising with its recent usage diversification from pharmaceutical, perfume industry to aromatic remedies. However, chemicals responsible for fragrance allergy have hardly been identified because its component is complex and its sensitization is not frequent. This report will present a case of acute allergic dermatitis that is likely induced by 3-hexylthiophene, one of aromatic compounds often contained in fragrance substances. The case, who was a 27-year male researcher engaged in organic chemical synthesis for six years, was exposed to 3-hexylthiophene and its product (2-bromo-3-hexylthiophene) through an experiment in May 2004 and itching, swelling and eczema immediately developed from face to back. This case of sensitization to 3-hexylthiophene suggests that it be a possible allergen for fragrance allergy.

## Background

Occupational cases of allergic dermatitis caused by aromatic compounds have been seen in the perfume industry and among aromatherapists[1,2]. Sensitization to aromatic compounds, although infrequent[3,4], has been reported sporadically since the 1970s as allergies to rubber products[5], anti-epileptic drugs[6], fragrance substances[7,8], and chemicals used in organic chemistry[9]. Common features of allergy to these are dermatitis on the axillae, face, neck, wrists, and behind the ears and hand eczema[3]. However, it has been difficult to identify the responsible chemicals from aromatic compounds because of their complex contents.

In these settings, volatile odorous mixtures of aromatic compounds are generally used as essential and fragrant oils. These oils often contain 3-hexylthiophene[1,10], which is also an aromatic compound. Whilst 3-hexylthiophene has recently been used to produce conducting polymers, major occupational sites of exposure to 3-hexylthiophene are the perfume industry and aromatherapy[1,2], where the number of workers using these oils is increasing[1,4]. We experienced a case of atopic dermatitis due to 3-hexylthiophene, which has not been identified as a cause of acute dermatitis.

## Case presentation

### Case story

The patient was a 27-year-old male researcher who had conducted research on organic chemical synthesis for six years. Several years ago, he developed eczema acutum on his face and neck during an experiment examining the synthesis of organic chemicals using ferrocene (CAS number 102-54-5). He consulted a dermatologist in a university hospital and was diagnosed with mild atopic dermatitis due to chemical exposure; however, the sensitizer was not identified in a multiple antigen test. He had no other history of dermatological disease. In May 2004, he conducted an experiment to examine the bromination of 3-hexylthiophene (CAS number 1693-86-3). The experiment is described in detail elsewhere[11]. He had performed the same experiment at a smaller scale approximately two weeks before this episode, but had not suffered from any dermatitis.

On the morning of 14 May 2004 (day 1), he dissolved 3-hexylthiophene (95 mmol) in 150 ml of chloroform (CAS number 67-66-3) and acetic acid (CAS number 64-19-7) in a 1:1 ratio by volume. The catalyst N-bromosuccinimide (95 mmol; CAS number 128-08-5) was added within 30 minutes with stirring. This process was conducted in a fume hood at room temperature, while the original experiment was performed at 0°C[11]. The solution was stirred for 30 minutes. After he extracted the products using a separating funnel in the afternoon, the extract was washed with KOH solution (2 mol) and diluted water, and then dried using a rotary evaporator. The extraction and evaporation of solvent were performed outside the fume hood for 30 minutes, although a stopper was used for this operation. Immediately during this process, itching and swelling spread from the periocular skin over his entire face. Throughout the experiment, he wore gloves, a laboratory coat, and goggles, and noticed no odours. In the same laboratory, another researcher conducted another organic synthesis experiment, but had no symptoms. The latter experiment was conducted inside a different fume hood that was sufficiently distant from the case to avoid exposure. By midnight, the rash and itching had spread over his entire body. There were no systemic symptoms such as vomiting, diarrhoea, dyspnoea, or wheezing.

On day 2, the chloroform was evaporated from the dry extract using a rotary evaporator. The extract was refined through a chromatography column filled with silica gel and hexane, producing 59.4 mmol (63%) of 2-bromo-3-hexylthiophene. Possible by-products of the experiment were other hexylthiophenes, such as 2-bromo-4-hexylthiophene, 3-bromo-4-hexylthiophene, 2,4-dibromo-3-hexylthiophene, 2,5-dibromo-3-hexylthiophene, 2,3-dibromo-4-hexylthiophene, and 2,4,5-tribromo-3-hexylthiophene. The volumes of these products were probably small.

**Figure 1:**
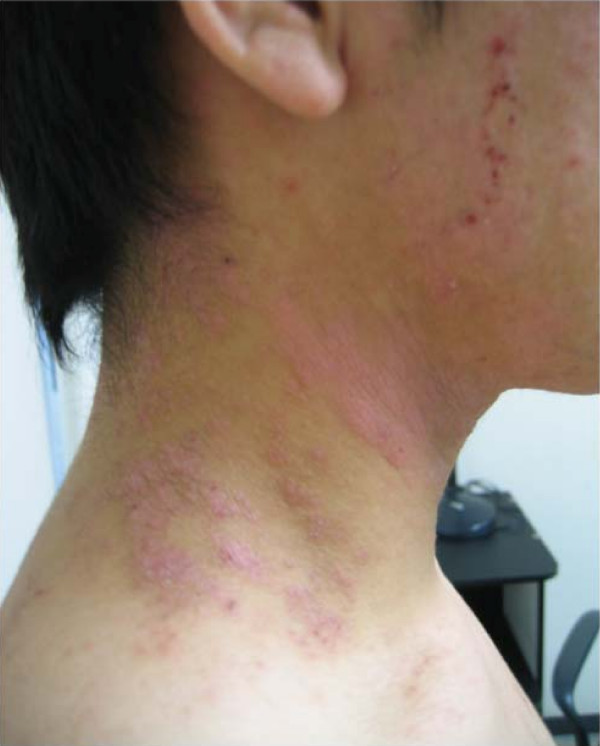
**Eczema on the patient's neck photographed on day 5**.

**Figure 2:**
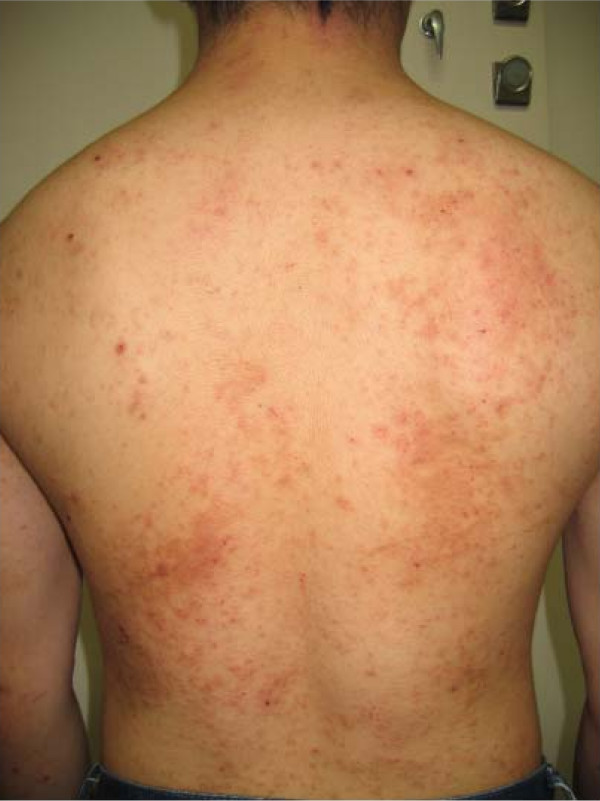
**Eczema on the patient's back photographed on day 5**.

The experiment was terminated on Day 3. His skin symptoms worsened further (Figures 1 and 2). On Day 8, a dermatologist diagnosed them as atopic dermatitis. They were treated with an ointment and anti-allergic pills beginning on Day 8. The eczema disappeared around Day 14, and he did not conduct any further experiments for one month. No patch test or scratch test was administered to detect a sensitizer. Since 2007, he has conducted similar experiments using these chemicals, except 3-hexylthiophene. No further dermatitis has developed.

## Discussion

Except for the extraction process on Day 1, the experiment was conducted in a fume hood with sufficient ventilation (> 0.5 *m*/*s*). For protection, the patient had worn gloves, a laboratory coat, and goggles. Nonetheless, eczema acutum developed rapidly on his neck and back immediately after he worked outside the fume hood on Day 1. It is postulated that a small amount of vapour of either the reagents used or the substances generated during the experiment[12] (Table 1) caused his acute dermatitis.

**Table 1:** Properties of the materials used or generated during the extraction process [2].

	Boiling point, °C	Flash point °C	Melting point °C	Skin irritation	Odor
N-Bromosuccinimide	NA	NA	180-183	+	+
3-Hexylthiophene	65	37	NA	+	+
2-Bromo-3-hexylthiophene	NA	110	NA	NA	+
Chloroform	62	NA	-64	+	+
Acetic acid	118	39	16.7	+	+

NA: Not available

The likely aetiology of the acute dermatitis in this case was atopic dermatitis or irritant contact dermatitis. Eczema may result from systemic, medication-induced, physical, or psychological causes or xerosis or infections[13]. However, these were unlikely because he did not have a history of any of these diseases.

For a definitive diagnosis, a scratch test or patch test is required, but they were not administered. We had to rely on deductive inference instead. The rapid development of eczema on his face and neck suggested either atopic dermatitis due to type 1 hypersensitivity or irritant contact dermatitis initially. The subsequent spread of the rash and itching to his entire body, where direct contact with any vapour was unlikely, indicated atopic dermatitis due to type 4 hypersensitivity as well. The rash on his skin was diagnosed as atopic dermatitis by a dermatologist. It is likely that the main aetiology was atopic dermatitis[14], although irritant contact dermatitis[15] may have preceded it.

3-Hexylthiophene, chloroform, and acetic acid evaporate readily at room temperature[12] and he may have been exposed to any of these. After this episode, however, he frequently used chloroform, acetic acid, and N-bromosuccinimide in other organic syntheses without developing a rash. Thus, it was unlikely that these chemicals, other than 3-hexylthiophene, were responsible for the patient's acute dermatitis.

## Conclusions

Occupational cases of allergic dermatitis caused by aromatic compounds have been seen in the perfume industry and among aromatherapists[1,2]. However, it remains difficult to identify the chemicals responsible of dermatitis from the complex contents of volatile oils[3,10,16]. This case suggests that hypersensitivity to 3-hexylthiophene be a possible cause of allergic dermatitis induced by volatile oils.

## Competing interests

The authors declare that they have no competing interests.

## Authors' contributions

MS gave aethiological consideration, reviewed relevant literature, and drafted the namuscript in English. HY drafted the manuscript in Japanese. MH reported the case. EY gave supervision on this report from a perspective of occupational hygiene. All authors read and approved the final manuscript.

## Consent

The patient approved the publication of this episode, but would not consent to any invasive procedure to obtain a definitive diagnosis. A copy of the written consent is available for review by the editor-in-chief of this journal.

## References

[B1] Dharmagunawardena B Takwale A Sanders KJ Cannan S Rodger A Ilchyshyn A Gas chromatography: an investigative tool in multiple allergies to essential oils Contact Dermatitis 2002 47 288 292 10.1034/j.1600-0536.2002.470506.x 12534533

[B2] Keane FM Smith HR White IR Rycroft RJ Occupational allergic contact dermatitis in two aromatherapists Contact Dermatitis 2000 43 49 51 10902596

[B3] de Groot AC Frosch PJ Adverse reactions to fragrances. a clinical review Contact Dermatitis 1997 36 57 86 10.1111/j.1600-0536.1997.tb00418.x 9062742

[B4] Paulsen E Contact sensitization from Compositae-containing herbal remedies and cosmetics Contact Dermatitis 2002 47 189 198 10.1034/j.1600-0536.2002.470401.x 12492516

[B5] Rudzki E Pattern of hypersensitivity to aromatic amines Contact Dermatitis 1975 1 248 249 10.1111/j.1600-0536.1975.tb05402.x 1235262

[B6] Conilleau V Dompmartin A Verneuil L Michel M Leroy D Hypersensitivity syndrome due to 2 anticonvulsant drugs Contact Dermatitis 1999 41 141 144 10.1111/j.1600-0536.1999.tb06105.x 10475512

[B7] Patlewicz GY Wright ZM Basketter DA Pease CK Lepoittevin JP Arnau EG Structure-activity relationships for selected fragrance allergens Contact Dermatitis 2002 47 219 226 10.1034/j.1600-0536.2002.470406.x 12492521

[B8] Sugiura M Hayakawa R Kato Y Sugiura K Hashimoto R Results of patch testing with lavender oil in Japan Contact Dermatitis 2000 43 157 160 10.1034/j.1600-0536.2000.043003157.x 10985632

[B9] Fowler JF Edge JC Occupational airborne allergic contact dermatitis from succinimidyl carbonates Contact Dermatitis 2001 45 38 10.1034/j.1600-0536.2001.045001038.x 11422267

[B10] Williams JD Tate BJ Occupational allergic contact dermatitis from olive oil Contact Dermatitis 2006 55 251 252 10.1111/j.1600-0536.2006.00916.x 16958929

[B11] Higuchi H Nakayama T Koyama H Ojima J Wada T Sasabe H Synthesis and properties of α, ω-disubstituted oligo (3-hexylthiophene)s and oligothienoquinonoids in head-to-head orientation Bull Chem Soc Jpn 1995 68 2363 2377 10.1246/bcsj.68.2363

[B12] Sigma-Aldrich Structure Search http://www.sigmaaldrich.com/chemistry/chemical-synthesis/chemical-synthesis-catalog.html

[B13] Rakel RE Common dermatologic symptoms. In: Saunders Manual of Medical Practice 1996 Philadelphia, London, Toronto, Montreal, Tokyo. W.B.Saunders 908 918

[B14] Rakel RE Atopic dermatitis, Contact dermatitis. In: Saunders Manual of Medical Practice 1996 Philadelphia, London, Toronto, Montreal, Tokyo. W.B.Saunders 919 920

[B15] Rakel RE Contact dermatitis. In: Saunders Manual of Medical Practice 1996 Philadelphia, London, Toronto, Montreal, Tokyo. W.B.Saunders 924 925

[B16] Trattner A David M Lazarov A Occupational contact dermatitis due to essential oils Contact Dermatitis 2008 58 282 284 10.1111/j.1600-0536.2007.01275.x 18416758

